# Microglia Morphological Changes in the Motor Cortex of hSOD1^G93A^ Transgenic ALS Mice

**DOI:** 10.3390/brainsci11060807

**Published:** 2021-06-18

**Authors:** Sara Migliarini, Silvia Scaricamazza, Cristiana Valle, Alberto Ferri, Massimo Pasqualetti, Elisabetta Ferraro

**Affiliations:** 1 Department of Biology, University of Pisa, 56126 Pisa, Italy; sara.migliarini@unipi.it (S.M.); massimo.pasqualetti@unipi.it (M.P.); 2 National Research Council, Institute of Translational Pharmacology (IFT), 00133 Rome, Italy; silviascaricamazza@gmail.com (S.S.); cristiana.valle@cnr.it (C.V.); alberto.ferri@cnr.it (A.F.)

**Keywords:** microglia, amyotrophic lateral sclerosis, neuroimmunology, metabolic reprogramming, SOD1G93A, motor cortex

## Abstract

Amyotrophic lateral sclerosis (ALS) is characterized by the progressive degeneration of spinal motor neurons as well as corticospinal (CSN) large pyramidal neurons within cortex layer V. An intense microglia immune response has been associated with both upper and lower motor neuron degeneration in ALS patients, whereas microgliosis occurrence in the motor cortex of hSOD1^G93A^ mice—the best characterized model of this disease—is not clear and remains under debate. Since the impact of microglia cells in the neuronal environment seems to be crucial for both the initiation and the progression of the disease, here we analyzed the motor cortex of hSOD1^G93A^ mice at the onset of symptoms by the immunolabeling of Iba1/TMEM119 double positive cells and confocal microscopy. By means of Sholl analysis, we were able to identify and quantify the presence of presumably activated Iba1/TMEM119-positive microglia cells with shorter and thicker processes as compared to the normal surveilling and more ramified microglia present in WT cortices. We strongly believe that being able to analyze microglia activation in the motor cortex of hSOD1^G93A^ mice is of great importance for defining the timing and the extent of microglia involvement in CSN degeneration and for the identification of the initiation stages of this disease.

## 1. Introduction

Amyotrophic lateral sclerosis (ALS) is a devastating neurodegenerative disease characterized by degeneration of the motor neuron circuitry; both spinal/bulbar (lower) motor neurons (SMN) and motor cortex corticospinal/corticobulbar (upper) neurons (CSN-Betz cells in humans) are affected. This is associated with progressive skeletal muscle atrophy and weakness, followed by complete paralysis and death [1].

Based on the corticofugal hypothesis, the motor cortex represents a relevant player in disease initiation, as the pathology seems to propagate from upper motor neurons to their downstream targets [2,3]. Indeed, cortical hyperexcitability is typical of ALS patients and has been proposed to lead to glutamatergic excitotoxicity to downstream targets such as SMN, thus providing a possible mechanism for disease propagation [4]. This might be associated with a loss of inhibitory GABAergic interneurons in the primary motor cortex, which might account for hyperexcitability [5]. Notably, motor cortex hyperexcitability has been recorded prior to disease onset, and TDP-43 aggregates—another pathological hallmark of the disease—are mainly found in the motor cortex in post-mortem specimens, all features suggesting a primary cortical involvement in disease initiation [6,7]. 

Although the involvement of the motor cortex, with degeneration of CSN large pyramidal neurons within layer V and related subcerebral projection neurons, has less frequently been reported in mouse models of disease compared to humans [5], some studies strongly suggest that the mouse models which best mimic ALS symptoms also recapitulate CSN degeneration [8,9,10,11].

Transgenic mice overexpressing the G93A mutated form of the human copper–zinc superoxide dismutase (SOD1) gene (hSOD1^G93A^ mice) present one of the best-characterized models for ALS [12,13]. hSOD1 mutations are toxic “gain of function” mutations, accounting for about 10–20% of familial ALS. hSOD1^G93A^ mice display an ALS-like SMN degeneration phenotype which has been widely analyzed, whereas CSN alterations, typical of ALS patients, have been less studied [14]. Several reports describe the presence of neuropathological alterations in hSOD1^G93A^ mice at a spinal–bulbar level. In addition, more recent studies have clearly identified an involvement of the corticospinal tract in hSOD1^G93A^ mice, with CSN loss and related subcerebral projection neurons undergoing early neurodegeneration [8,9,15,16,17]. A reduction in calretinin-positive interneurons, as well as hyperexcitability of layer V pyramidal neurons typical of ALS patients, has also been observed in the cerebral cortex of hSOD1^G93A^ mice [18,19]. Moreover, a similar mouse model of ALS with SOD1 bearing a different mutation—SOD1^G86R^ mice—displays presymptomatic CSN degeneration and a somatotopic relationship between CSN and spinal motor neuron degeneration, as typically reported in ALS patients [20]. 

Motor neuron degeneration in humans is associated with astrogliosis and intense innate immune responses of microglia (microgliosis), which have also been clearly demonstrated in the spinal cord of mouse models of ALS [21,22,23,24,25,26,27]. However, although the occurrence of microgliosis in the diseased motor cortex and the interaction of activated microglia with degenerating Betz cells has been revealed in postmortem samples and by imaging studies in ALS patients [15,28,29,30], the activation of microglia in the cortex of hSOD1^G93A^ mice has not been clearly demonstrated [9,15,17,31,32]. To sum up, the timing and extent of microglia activation in ALS remains under debate; do microglia contribute to motor neuron degeneration, or is it a by-product of neuronal death?

Microglia cells undergo different activation states by switching from a mature “surveilling” state, typical of physiological brain parenchyma, to an “activated” state, during which they change their sensome and their secretome, as well as their metabolism, and gradually modify their morphology from a highly branched shape with a small soma and very fine long processes into a less ramified or ameboid morphology [33,34,35,36,37,38]. In fact, besides the increase in microglia cell numbers, evaluation of morphological features represents a key marker for microglial activation and functional state [39]. Between the two extremes of ramified and amoeboid shapes—in “surveilling” and in “activated” subsets, respectively—microglia display a broad range of morphological changes revealing the intricate phenotypic modifications occurring during their activation in vivo [27,40,41]. 

Although ALS is caused by the progressive degeneration of motor neurons, the impact of non-neuronal cells in the neuronal environment seems to be crucial for both the initiation and the progression of the disease. Here, we analyze the hSOD1^G93A^ mice motor cortex neuronal environment by specifically focusing on microglia in order to investigate if widely distributed non-neuronal pathology is recapitulated in hSOD1^G93A^ mice. Confocal immunolabeling techniques are used to mark and monitor the cells of interest.

## 2. Materials and Methods

### 2.1. Animals

All animal procedures were approved by the Animal welfare office, Department of Public Health and Veterinary, Nutrition and Food Safety, General Management of Animal Care and Veterinary Drugs of the Italian Ministry of Health (protocol number 931/2017/PR) and carried out in agreement with European guidelines for the use of animals in research (2010/63/EU) and the requirements of Italian laws (D.L. 26/2014). Animals were kept in a virus/antigen-free facility with a light/dark cycle of 12 h at constant humidity and temperature and with food/water ad libitum. hSOD1^G93A^ mice (B6.Cg-Tg(hSOD1^G93A^)1Gur/J) were obtained from The Jackson Laboratory (Bar Harbor, ME, USA) and bred in our animal facility; transgenic hemizygous hSOD1^G93A^ males were crossbred with C57BL/6 females and transgenic progeny were genotyped by PCR. The disease onset was evaluated by the hanging grip test, as previously described [42]. Briefly, we measured how long the mouse was able to grasp the grid; we started the measurement at 50 days of age (considered as 100%) and we performed it twice a week until the mouse reached 20% of the performance, which we considered a failure of the test (Figure S1A). By this method, we detected the onset of disease at 91.7 ± 2.9 days of age. To monitor disease progression, we recorded the body weight (Figure S1B) and the behavioral scores (Figure S1C), as previously described [43]. 

### 2.2. Immunohistochemistry Analysis

Animals were deeply anesthetized (avertin, i.e., 250 mg/kg) and transcardially perfused with phosphate-buffered saline, followed by 4% paraformaldehyde (PFA). Brains were dissected and post-fixed overnight at 4 °C in 4% PFA. Coronal sections of 100 µm were obtained with a vibratome (Leica Microsystems, Milan, Italy). Immunohistochemical procedures were performed on free-floating sections, as described in Pratelli et al. [44]. Briefly, sections were incubated overnight at 4 °C with a goat anti-Iba1 (1:1000, Abcam, Cambridge, UK) and a rabbit anti-TMEM119 (1:1000, GenTex, Irvine, CA, USA) primary antibody, followed by incubation overnight at 4 °C with a Donkey α Goat Alexa Fluor 488 for Iba1 and Donkey α Rabbit Alexa Fluor 594 for TMEM119 (1:500, Life Technologies, Monza, Italy). Cell nuclei were counterstained with 4’,6-Diamidin-2-phenylindol (DAPI, Merck Life Science, Milan, Italy), 0.5 mg/mL in PBS. Images were acquired using a Nikon-A1 confocal microscope. 

### 2.3. Image Analysis and Quantification

Quantification analyses were performed blind, and sample identity was not revealed until correlations were completed. For the quantification of the number of glial cells in the motor cortex of hSOD1^G93A^ and control animals, Iba1 and TMEM119 double positive cells were counted on 5 consecutive coronal sections throughout the rostro-caudal extent of motor cortex layer V. Z-stack confocal images (21 stacks) were acquired at 0.5 μm intervals using a 20× Nikon objective. Consecutive Z-stack images were converted to a maximum intensity projection and the ImageJ Cell Counter analysis plugin was used. In order to evaluate the morphological complexity of glia cells in the motor cortex of hSOD1^G93A^ animals, manual morphological analysis was performed using FIJI software (Version 2.0) and the Sholl analysis plugin [45]. Z-stack confocal images (69 stacks) were acquired at 0.15 μm intervals using a 60× Nikon objective. Consecutive Z-stack images were converted to a maximum intensity projection image using FIJI software, and individual cells positive for Iba1 and TMEM119 were identified. For each cell, the center of the soma was set using centroid function of the software and the region of interest (ROI) of the cell was manually drawn. Maximum intensity projections of the cells were thresholded for creating a binary mask. For the Sholl analysis, the number of intersections was calculated, starting from 5 μm from the center of the soma, and with a radius step size of 2.5 μm. For each animal, a minimum of 4 cells for 5 consecutive coronal sections throughout the rostro-caudal extent of motor cortex layer V were analyzed. 

### 2.4. Statistical Analysis

Data are expressed as mean ± SEM, and tests of significance were conducted using Student’s *t*-tests and Multiple *t*-tests. The *p*-values resulting from statistical tests are indicated. GraphPad Prism was used for all analyses.

## 3. Results

### 3.1. Motor Cortex Microglia Cell Number Does Not Change in hSOD1^G93A^ Mice Compared to WT Animals

**Figure 1:**
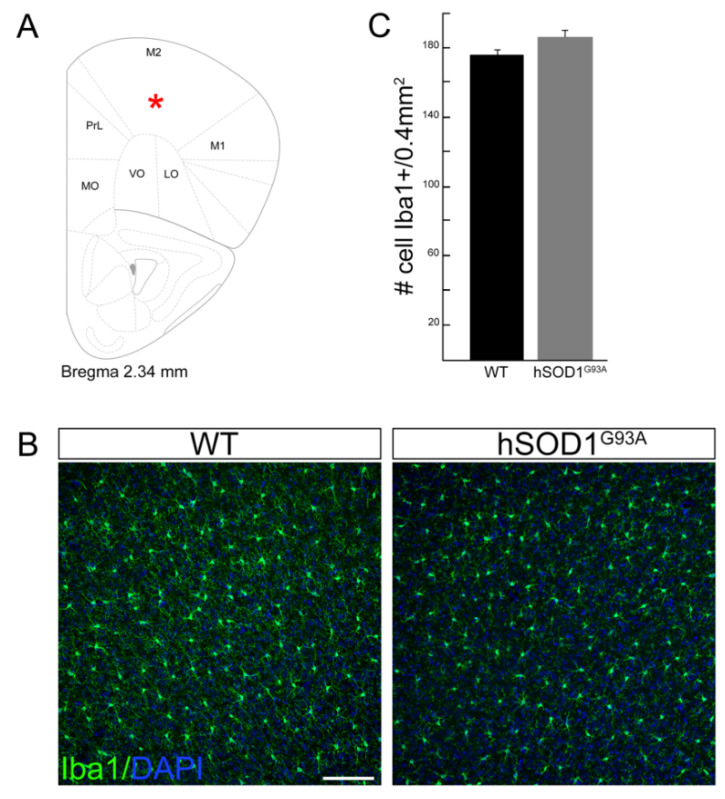
(**A**) Coronal table adapted from the mouse brain atlas [46], showing the anatomical localization of the analyzed region (motor cortex layer V; asterisk) and corresponding to the high magnification images in (**B**). (**B**) Representative images of motor cortex layer V coronal sections showing Iba1 immunoreactivity in 90-day-old WT and hSOD1^G93A^ mice. (**C**) Bar graph showing the average number of Iba1-positive cells in 5 consecutive coronal sections (0.4 mm^2^ each) of hSOD1^G93A^ motor cortices as compared to WT ones (*n* = 3 mice per genotype). Data are presented as mean ± SEM. Scale bar: 100 μm. M2, secondary motor cortex; PrL, prelimbic cortex; M1, primary motor cortex; MO, medial orbital cortex; VO, ventral orbital cortex; LO, lateral orbital cortex.

As the occurrence and the features of cortical microgliosis in the ALS mouse model hSOD1^G93A^ remain an open question, we performed a confocal analysis of motor cortex microglia cells in WT and hSOD1^G93A^ mice at 90 days of age (onset of symptoms) by immunolabeling of the microglial marker ionized calcium-binding adaptor molecule 1 (Iba1). We performed our analysis on brain coronal sections, focusing on layer V of the motor cortex (Figure 1A). In line with data concerning the motor cortex of SOD1 mutation-bearing ALS patients in which the overall number of microglia cells does not change [15], we did not observe a different number of Iba1-positive cells in hSOD1^G93A^ transgenic mice compared to WT ones (WT 175.1 ± 3.7 vs. 185.7 ± 5.8 in hSOD1^G93A^; Figure 1B,C). 

This might suggest an absence of a microglia-specific inflammatory response occurring in the motor cortex of this ALS mouse model, which would be different from what occurs in the spinal cord of the same mouse model [21].

### 3.2. Morphological Complexity Reduction in Iba1/TMEM119-Positive Microglial Cells in Motor Cortex of hSOD1^G93A^ Mice 

Although the number of microglia cells in the cortex of 90-day-old hSOD1^G93A^ mice appeared unaffected, we could observe that each microglia cell seemed to be less ramified compared to WT microglia (Figure 1B). Therefore, we then went into more detail with the purpose of investigating a potential shift in microglia activation state not associated to cell proliferation. We analyzed the morphology of Iba1-positive cells to distinguish between “normal surveilling” microglia, defined by a typically ramified appearance with fine processes, and “activated” microglia, defined by shorter and thicker processes [39].

**Figure 2:**
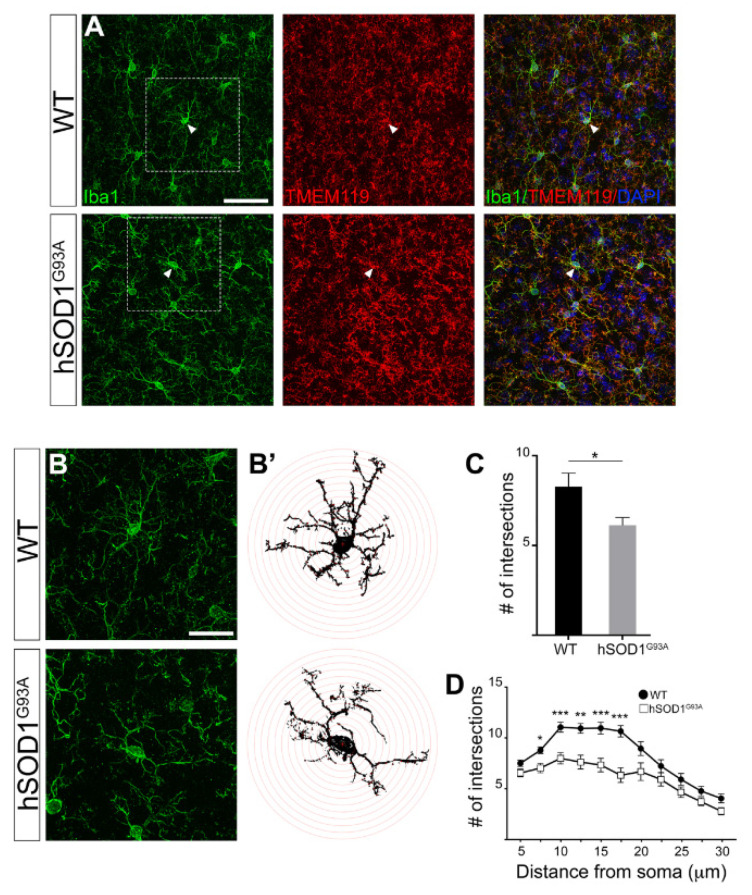
(**A**) Representative confocal images of motor cortex layer V coronal sections showing Iba1 and TMEM119 immunoreactivity in WT and hSOD1^G93A^ adult mice. Arrowheads point to the soma of the cells. (**B**) High magnification images of the boxed regions in (**A**, Iba1) and the relative Sholl mask with concentric Sholl radii (red circle on right panels; **B’**) of single microglia cells. (**C**) Bar graph of the average of the total number of ramification intersections of microglia cells with all concentric circles (*n* = 3 mice per genotype, 20 cells per mouse). The data show a significant decrease in the total number of intersections in microglia cells of hSOD1^G93A^ mice as compared to WT ones. (**D**) Sholl analysis plot showing a significant decreased number of ramifications at 7.5–17.5 µm from the soma in microglial cells of hSOD1^G93A^ mice as compared to WT ones. Scale bar: 50 μm (**A**) and 24 μm (**B**).

Since it has recently been proposed that Iba1 is also expressed by infiltrating macrophages in the central nervous system (CNS) [47,48], in order to specifically evaluate microglia in the motor cortex of hSOD1^G93A^ mice, we utilized the more specific microglia marker transmembrane protein 119 (TMEM119) [48,49]. TMEM119 staining revealed an unprecedented visualization of microglia cells in the motor cortex of hSOD1^G93A^ mice. Therefore, to specifically focus on microglia cells, thus excluding CNS infiltrating macrophages which are also present in ALS brains, we focused our analysis on Iba1/TMEM119 double positive cells. All Iba1 ^+^ cells we detected and analyzed were also TMEM119^+^ (Figure S2); this might be due to the fact that—as previously suggested in [50]—peripheral macrophage infiltration is not yet present in the cortices of 90-day-old hSOD1^G93A^ mice. Therefore, given that all Iba1^+^ cells were also TMEM119-positive (Figure S2), by counting them we could confirm that the number of Iba1 ^+^ cells reported in Figure 1 also corresponded to the number of Iba1/TMEM119 double positive cells. We studied Iba1 staining in Iba1/TMEM119 double positive cells by confocal microscopy, and in the hSOD1^G93A^ motor cortex microglia we observed a lower morphological complexity with fewer secondary ramifications—typical of activated microglia—as compared to WT mice (Figure 2A).

To evaluate the extent of our observation and quantify arborization in Iba1/TMEM119 double positive cells, we performed a Sholl analysis on the Iba1 labeling of double positive cells. Sholl analysis is a method widely used in neurobiology to quantify the complexity of dendritic arbors (Figure 2B–D). This quantification clearly confirmed that, in the motor cortex of 90-day-old hSOD1^G93A^ mice, microglia cells display a significantly reduced complexity, thus suggesting an activated phenotype compared to WT mice (Figure 2C,D). In more detail, we could also observe that the lower arborization complexity of Iba1/TMEM119-positive cells in hSOD1^G93A^ mice compared to WT ones is mostly evident in the region 10–17 μm away from the cell soma, whereas this difference disappears at longer distances (Figure 2D). 

**Figure 3:**
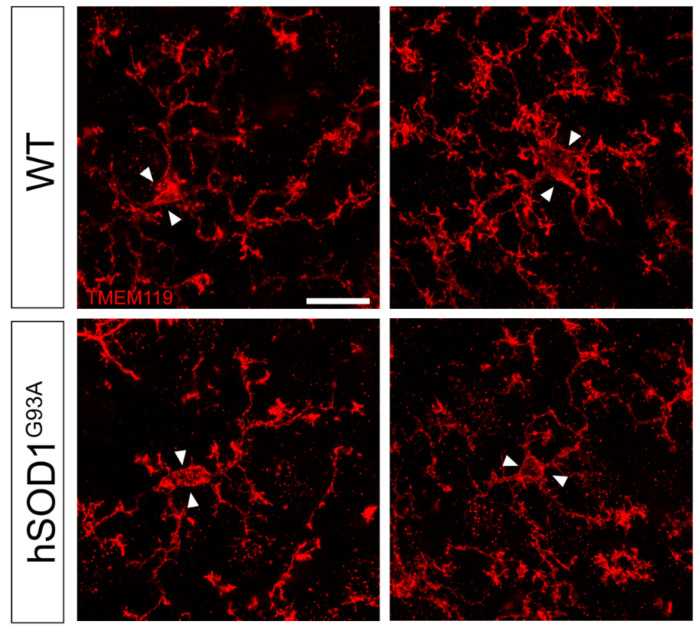
Representative high-magnification confocal images of motor cortex layer V coronal sections showing TMEM119 immunoreactivity in WT and hSOD1G93A 90-day-old mice and displaying a reduced microglia morphological complexity in hSOD1G93A mice compared to WT animals. Arrowheads point to the soma of cells. Scale bar: 15 μm.

We finally focused on TMEM119 staining, which revealed a further degree of cell complexity compared to Iba-1 staining, since more secondary ramifications and distal processes are highlighted. High magnification of TMEM119 staining showed a striking difference in the arborization complexity of motor cortex microglia cells between the two genotypes; hSOD1^G93A^ microglia display fewer ramifications and collaterals and more atrophic processes compared to TMEM119-positive cells in WT mice, thus paralleling and emphasizing the data obtained by Iba-1 staining (Figure 3).

## 4. Discussion

Although the role of microglia in the spinal component of the motor neuron circuitry in hSOD1^G93A^ mice has been widely reported, its occurrence in the motor cortex is under debate. Through our experiments, we were able to identify the presence of morphologically altered and fewer ramified Iba1/TMEM119-positive cells suggestive of activated microglia in the motor cortex of hSOD1^G93A^ mice at the onset of symptoms (90 days of age). The lower complexity and arborization of these cells might be explained by either the retraction of branches—typical of microglia activation—or by the fragmentation of branches, which would account for dystrophic microglia. Although the scattered and uneven localization pattern of TMEM119 would support this latter hypothesis, conversely, the continuous localization pattern of Iba1—in the same regions where TMEM119 is present—strongly supports the hypothesis of microglia activation while discarding the one of cell fragmentation.

The co-staining of Iba1 and TMEM119 guarantees the ability to distinguish bona fide microglia from infiltrating macrophages and, in our experiment, showed that, at this stage of disease, the number of motor cortex microglia cells was unchanged in hSOD1^G93A^ mice vs. WT ones, although an increasing trend was present. This suggests the utility of future analyses to evaluate the number of Iba1/TMEM119 double positive cells at later symptomatic stages of disease. Conversely, we were able to identify a diverse extent of morphological complexity among Iba1-TMEM119 double positive cells in hSOD1^G93A^ vs. WT motor cortices. Quantitative evaluation of such complexity, by means of Sholl analysis, revealed a statistically significant reduction in microglia cell arborization in hSOD1^G93A^ mouse cortices compared to WT ones. This suggests the presence of activated microglia, characterized by shorter and thicker processes compared to normal surveilling microglia, which are also more ramified. Moreover, TMEM119 staining revealed more distal processes compared to Iba1, and, although the uneven distribution of TMEM119 labeling on microglia branches prevented its quantification by Sholl analysis, a reduction in this other level of arborization was also highlighted by the TMEM119 immunostaining. Our observation is a starting point which supports the need for further studies on this topic in order to confirm and elucidate microglia activation features by the analysis of specific markers at various stages of disease [51].

## 5. Conclusions

Given that the motor cortex is involved in the first stages of ALS and that the role of microglia is crucial in this disease, we strongly believe that being able to analyze motor cortex microglia activation in hSOD1^G93A^ mice—the most studied mouse model of ASL—is of great importance for identification of the precise nature of the primum movens in this disease. This tool will make a helpful contribution to defining the timing and the extent of microglia involvement in CSN degeneration as well as neuroimmune interactions such as macrophages and T-cell recruitment to sites of injury, all of which remain under debate in ALS. Finally, revealing the various facets of the contribution of the diseased cortex to ALS pathology is of great importance, since precocious pathophysiological and diagnostic biomarkers of cortical involvement might provide insights for the development of future therapeutic approaches in ALS. 

## Data Availability

The data presented in this study are available on request from the corresponding author.

## Supplementary Materials

The following are available online at https://www.mdpi.com/article/10.3390/brainsci11060807/s1, Figure S1: Disease onset of hSOD1^G93A^ mice based on hanging grip test and disease progression evaluation by body weight and behavioral score, Figure S2: Representative confocal images of microglia in motor cortex coronal sections showing Iba1 and TMEM119 immunoreactivity in WT and hSOD1^G93A^ 90-day-old mice.
