# Supplementary material for: Microglia Morphological Changes in the Motor Cortex of hSOD1G93A Transgenic ALS Mice
Source: Brain Sci. 2021 Jun 18;11(6):807. doi: 10.3390/brainsci11060807 (PMC8234003; doi:10.3390/brainsci11060807)
Supplement: Supplementary file 1 [file brainsci-11-00807-s001.zip › brainsci-1233247-Supplementary.pdf]

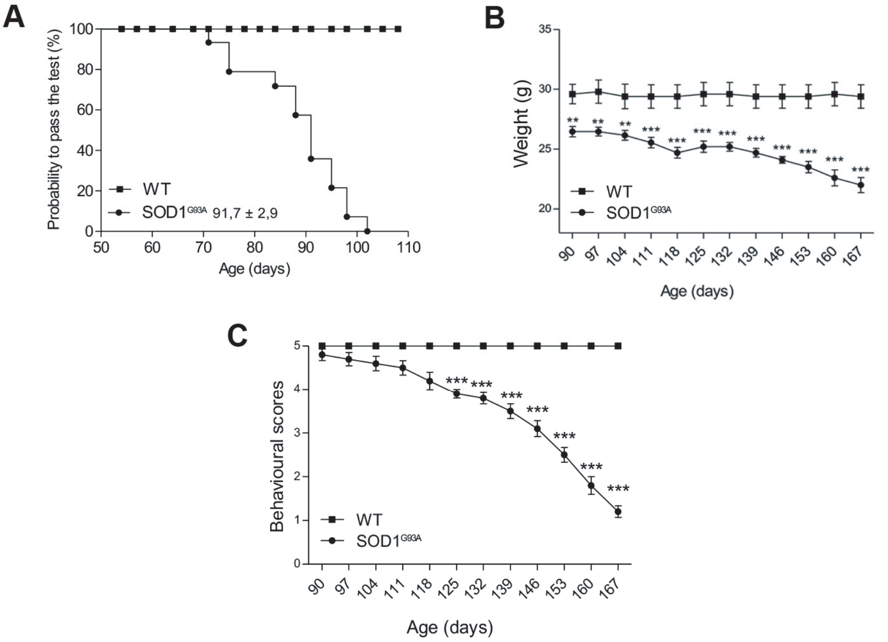

Figure S1. **A**) Kaplan-Meier representing the disease onset of SOD1G93A mice compared WT mice based on the hanging grip test (see Material and Methods). Weight (**B**) and behavioural score (**C**) evolution of SOD1G93A and WT mice. **p<0,01, ***p<0,0001. At least n=12/group. p Value was obtained using parametric two-way ANOVA and Bonferroni post hoc test.

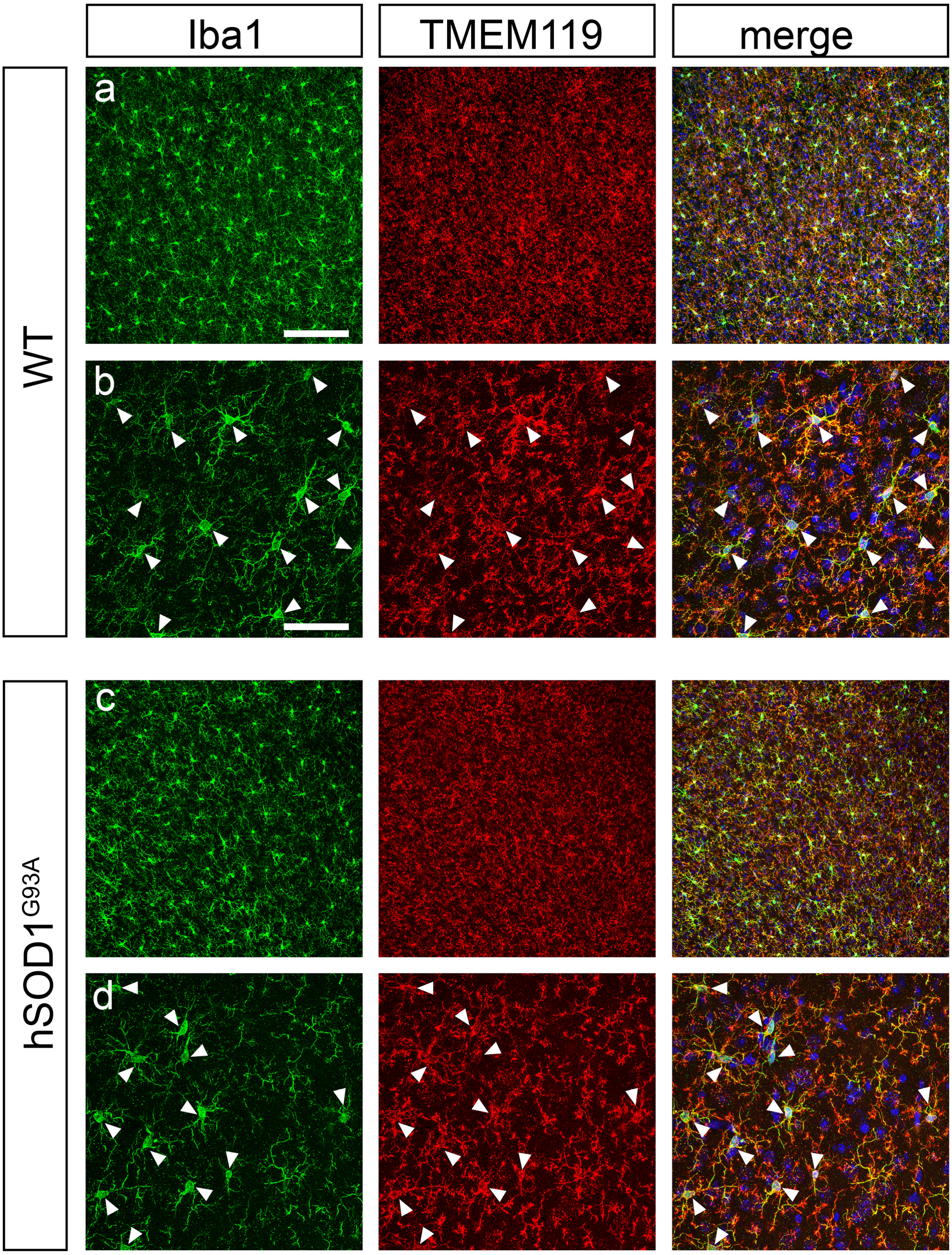

Figure S2. Representative confocal images of microglia in motor cortex coronal sections showing Iba1 and TMEM119 immunoreactivity in WT (**a-b**) and hSOD1G93A (**c-d**) 90-day-old mice. Arrowheads point to the soma of Iba1 and TMEM119 double labeled cells. Scale bar: 150 μm (**a,c**) and 50 μm (**b,d**).
